# CyberKnife radiation therapy for malignant myopericytoma in a pediatric patient: a case report and review of the literature

**DOI:** 10.1007/s00066-025-02413-z

**Published:** 2025-06-16

**Authors:** Bence Bukovszky, Júlia Vízkeleti, Levente Jánváry, Gábor Szarvas, Luca Felkai, Rita Bánusz, Edit Varga, Zoltán Sápi, Tibor Major, Monika Csóka

**Affiliations:** 1 Pediatric Center, Semmelweis University, Budapest, Hungary; 2 Radiotherapy Centre, National Institute of Oncology, Budapest, Hungary; 3 Medical Imaging Centre, Semmelweis University, Budapest, Hungary; 4 Department of Pathology and Experimental Cancer Research, Semmelweis University, Budapest, Hungary; 5 Department of Oncology, Semmelweis University, Budapest, Hungary; 6 National Tumour Biology Laboratory, National Institute of Oncology, Budapest, Hungary; 7 7-9. Tűzoltó Str., 1094 Budapest, Hungary

**Keywords:** Malignant myopericytoma, CyberKnife radiation therapy, Soft tissue tumors, Rare pediatric tumors, Inner and middle ear tumor

## Abstract

**Background:**

Malignant myopericytoma is a very rare malignant soft tissue tumor which usually develops during adulthood. It has a very poor prognosis due to its aggressive nature and frequent distant metastases.

**Methods:**

In our case report, we present a 17-month-old girl with malignant myopericytoma who was successfully treated using CyberKnife (Accuray; Sunnyvale, CA, USA) stereotactic radiotherapy.

**Results:**

A rare localization of the tumor caused significant challenges during the course of treatment. Radical surgical resection was not achievable due to the tumor’s location in the inner ear; therefore chemotherapy was initially given to the patient. However, due to the fast progression of the tumor during chemotherapy, we decided to use CyberKnife stereotactic radiosurgery (SRS; 5 fractions of 7 Gy) in order to prevent further invasion of the surrounding tissues. During SRS, tumor growth stopped and the tumor then gradually regressed. Since completion of treatment (currently almost 5 years) our patient has been in complete remission without any significant side effects of the radiation therapy.

**Conclusion:**

In our recent experience, systemic chemotherapy combined with CyberKnife SRS proved to be effective in a patient with a rare malignant myopericytoma.

## Introduction

Malignant myopericytoma was first described by Granter et al. in 1998 [1]. The WHO officially accepted the term myopericytoma in the pericyte group in 2002 (Classification of Tumors of Soft Tissue and Bone) [2]. According to the 2013 WHO classification, malignant myopericytoma belongs to the pericytic tumors [3].

Myopericytomas are rare tumors that appear mostly in the skin and superficial soft tissues. According to the literature, the most commonly affected locations are the limbs (upper arm, thigh, ankle) and the neck, but Mainville et al. reported a case of malignant myopericytoma originating from the heart [4]. Based on a comprehensive review of the literature existing to date, malignant myopericytoma is an extremely rare tumor, with the number of reported cases not exceeding 20 [4–10, 13]. Almost all reported cases were in adults, with only one case describing the disease in a 15-year-old patient [13]. Complete resection of the tumors was generally possible. In some cases, the surgical treatment was supplemented with chemotherapy or radiation therapy. Although the true benefits of chemotherapy and radiation therapy were not described, they should be considered due to the high likelihood of metastases and recurrence [8]. Overall, the prognosis of this disease is poor [5].

Our case report is unique in terms of the patient’s age, the tumor location, and the choice of treatment. However, due to the rarity of this tumor, no general conclusions can be drawn from the results.

## Methods and results

We herein present the case of a baby girl born in 2017, who, between the ages of 1 and 12 months, regularly suffered from a yellowish-white discharge from her left ear and was diagnosed several times with otitis media, resulting several antibiotic treatments.

**Fig. 1:**
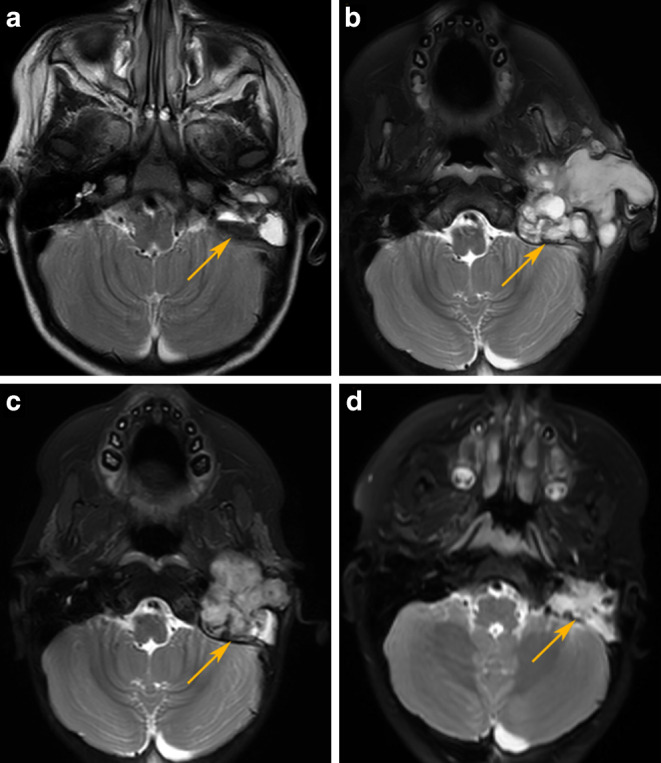
**a**–**d** Axial T2W images. **a** The initial MRI (June 2018) shows a cystic lesion in the left mastoid region with fluid–fluid level (arrow). At the first follow-up examination (**b**; July 2018) during active chemotherapy, in the T2 FS image, we can see 75% progression with the appearance of a solid tumor component as well. The next follow-ups (T2 FS images) show the tumor before radiation therapy (**c**; February 2019) and 4 years after radiation therapy (**d**; July 2023)

At 15 months of age, left-sided facial paresis developed. Therefore, a CT scan of the inner ear was performed, which showed a large soft tissue mass measuring 25 mm in the left external auditory canal, which was causing destruction of the auditory canal and middle ear. The mass also partially compressed the mastoid system, penetrating into the jugular foramen without brain involvement. According to the results of the head, middle ear, and neck MRI examination, the alterations visible in the left middle ear were more likely a consequence of an inflammatory process rather than tumorous mass (Fig. 1a). Due to the uncertain radiographic results, a biopsy was performed, which initially contained only epithelial and bone tissue.

**Fig. 2:**
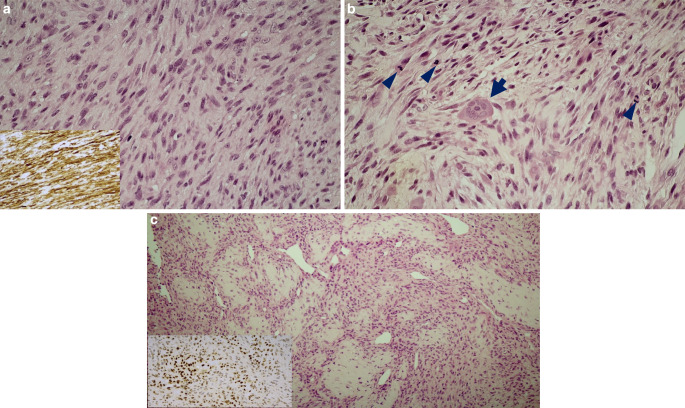
Compact cellular fascicles of atypical spindle cells; insert: strong alpha smooth muscle actin immunostaining (**a**). High mitotic activity (arrowheads) was characteristic, and scattered osteoclast-type giant cells (arrow) were also observed (**b**). Hemangiopericytic pattern with cystic dilated vascular gaps with associated hyaline/bone-like formation; however, osteoid produced by tumor cells was not observed; insert: strong focal SATB2 positivity (**c**)

The repeated biopsy confirmed a malignant myopericytoma (myopericytic sarcoma, grade II; Fig. 2). During initial staging examinations, no distant metastases or bone marrow or CNS involvement were detected.

During the overall literature review, we encountered an extremely rare entity that had never been described in such a young child before. Due to the malignant nature and rapid progression of the disease, we immediately started chemotherapy with a VAC course according to the CWS 2012 Soft Tissue Sarcoma Protocol. Two weeks after administration of the first systemic chemotherapy, a follow-up MRI examination was performed, in which 75% tumor progression was detected (Fig. 1b), and there was a suspicion of metastatic lymph nodes at the caudal end of the tumor and in the left supraclavicular region.

Due to the patient’s deteriorating respiratory status and difficulty in swallowing and feeding, tracheostomy and gastrostomy (PEG) were performed. Between August 2018 and February 2019, our patient received systemic chemotherapy according to the CWS 2012 Protocol’s metastatic, CEVAIE treatment arm. For the future, continuation of systemic chemotherapy according to the selected protocol followed by maintenance treatment was planned. Apart from the cytopenic episodes following chemotherapy blocks, no major complications or adverse events occurred. Follow-up MRIs were performed every 2 months, during which an initial 40% tumor regression followed by an additional 25% reduction in tumor size was seen. In contrast to earlier results, MRI performed in February 2019 showed 8% tumor progression (Fig. 1c). Due to the location and the extent of the tumor invasion, radical surgery was not possible. Since urgent local treatment was necessary, we decided to initiate radiation therapy. We did not find any recommendations for a similar location and histological type in the literature; thus, considering the very young age of the patient and the significant physical symptoms caused by tumor progression (left-sided facial paresis and hearing loss), we decided to deliver 35 Gy total dose in 5 fractions with 7‑Gy fractional doses using the CyberKnife (Accuray Inc., Sunnyvale, CA, USA) robotic stereotactic linear accelerator, taking into account the tolerance of the surrounding organs. CyberKnife is a stereotactic radiosurgery system consisting of a 6-MV linear accelerator and a robotic arm [11]. Using image guidance, the dose can be precisely delivered to tumors with many small noncoplanar radiation beams defined by multileaf collimators (MLCs). With frequent X‑ray imaging, patient positioning can be checked using the information of bone structures (skull), and, in the case of geometrical deviations, the robotic arm performs an automatic correction. With this technique, submillimeter accuracy of dose delivery can be maintained. Using MLCs, step-and-shoot intensity-modulated radiotherapy can be performed, providing a highly conformal dose distribution.

According to the CWS 2012 Protocol, maintenance therapy includes anthracyclines, which cannot be given during radiotherapy. Therefore, in our case, we needed to further modify the treatment plans by switching into the EpSSG RMS 2005 Protocol. The very-high-risk maintenance therapy was initiated in early March 2019, with intravenous vinorelbine and oral cyclophosphamide.

The patient received radiation therapy (RT) with five fractions between March 20 and 27, 2019.

**Fig. 3:**
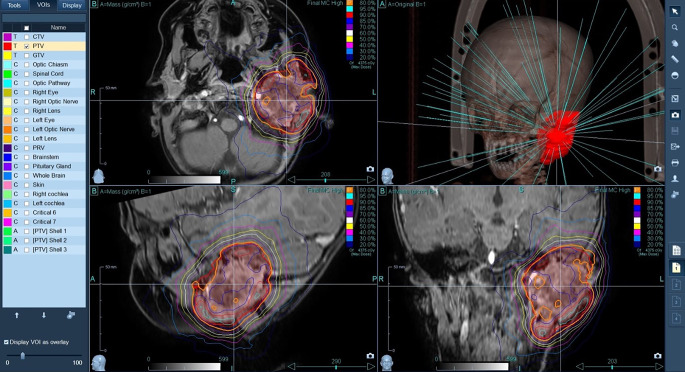
CyberKnife (Accuray Inc., Sunnyvale, CA, USA) treatment plan with relative isodoses (contours) and beam directions (blue lines) focusing on the tumor

Treatment planning was performed by CT. The pretreatment MRI images were fused with the planning CT scans to help to accurately define and delineate the target volume. For treatment planning, the Accuray Precision 3.3.1.2 (Accuray Inc.) planning system was used [12]. For dose calculation, a Monte Carlo algorithm was applied with 46 beams and 84 segments (Fig. 3). The prescribed dose of 35 Gy was given to the 80% isodose line, resulting in a maximal dose of 43.75 Gy (125%). The volume of the tumor was 31.3 cm^3^. A total safety zone of 3 mm around the tumor was applied to account for the possible microscopic spread of cancerous cells and the geometrical inaccuracy of patient setup. The volume of the resulting planning target volume was 47.7 cm^3^, and its mean dose was 37.7 Gy (108%). The mean dose to the left cochlea was 38.2 Gy (109%), while to the right cochlea it was only 2.45 Gy (7%). The maximal dose to the left and right optic nerves was 9.2 Gy (26%) and 10.5 Gy (30%), respectively. The optic chiasm received 4.7 Gy (13%) as the maximum.

Following RT, maintenance therapy was continued according to CWS 2012. The child received a total of four OTE and four OTI blocks. During this period, the follow-up MRI showed stable disease. In July 2019, left-sided cervical en bloc lymph node dissection was performed due to the risk of lymph node involvement (as suggested by MRI), in which the histological examination did not confirm any metastases. The tracheostomy and gastrostomy tubes were also removed successfully. The most recent MRI examination conducted in June 2024 showed an inactive residual lesion of unchanged size and smaller nasal and pharyngeal tonsils and submandibular salivary gland on the left side. Additionally, there was evidence of growth impairment in the mandible on the same side. These observed features correspond to post-radiation changes (Fig. 1d). No other long-term consequences of radiation therapy were detected.

**Fig. 4:**
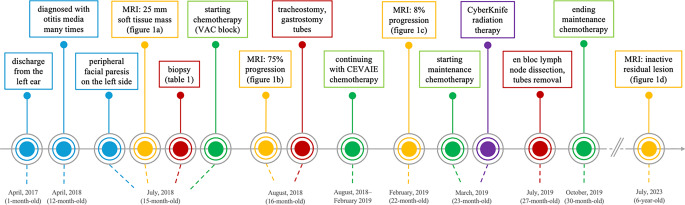
Flowchart of the medical history of the patient. Color coding: blue—symptoms, yellow—imaging, red—surgical interventions, green—chemotherapy, purple—radiation therapy

The flowchart of the medical history is illustrated in Fig. 4. The child now attends school and is doing well, with the only remaining symptom of hearing loss in the left ear and moderate peripheral facial nerve paresis, both of which were caused by the tumor before diagnosis.

## Discussion

Myopericytoma is an extremely rare entity belonging to the perivascular tumors, including myofibroma, angioleiomyoma, and glomus tumor. In clinical practice, the majority of myopericytomas develop in middle-aged adults and are generally considered benign, with malignant cases being extremely rare.

Myopericytoma refers to a group of tumors originating from perivascular myoid cells and showing several histological growth patterns. Based on both immunohistochemical and ultrastructural findings, myopericytoma is classified as a perivascular myoid cell tumor. It has characteristics of both smooth muscle cells and glomus cells. In its growth pattern, it shows some overlap with myofibromas. Myopericytes (and therefore myopericytoma) are formed from myofibroblast or pericyte cells, which have the properties of modified smooth muscle cells.

Unlike most solid tumors, soft tissue sarcomas develop from pluripotent mesenchymal stem cells that can differentiate in a certain direction, determining the behavior of the emerging tumor, including its response to therapy.

A correct histological diagnosis is essential for selecting the appropriate therapy (which usually consists of a combination of surgical resection, chemotherapy, and radiation therapy). Recently, immunohistochemical and molecular tests have been widely used to help make a more accurate diagnosis. By immunohistochemistry, myopericytomas usually show a desmin-negative, actin-positive phenotype [1, 2, 14].

In our case, histological examination showed that the tumor cells were elongated, spindle shaped, and formed irregular bundles. Vessels showed a hemangiopericytoma-like pattern in several places. No significant polymorphism or prominent nucleoli were observed, and more than 10 divisions per 10 high-power fields were seen in hotspot areas. Osteoclast-type giant cells were also detected next to the tumor cells. Immunoreactions showed diffuse vimentin and rather diffuse alpha-smooth muscle actin positivity. Desmin, Myf4, and H‑caldesmon reactions were all completely negative, excluding the possibility of rhabdomyosarcoma and leiomyosarcoma. Other negative reactions included CD34, S100, and ALK1. Ki67 showed a proliferation rate of approximately 25–30%. Diffuse positivity with p16 was found, but its significance in this tumor is unknown. FISH testing with ETV6, NTRK1, and NTRK3 break-apart probes showed no signal separation. The negativity of ETV6 and NTRK1 excludes the possibility of infantile fibrosarcoma. Even though NTRK1 was negative, the lesion can still be clearly considered a malignant myopericytoma based on its morphology. Additional immunostaining with SATB2 showed clear nuclear positivity in some of the tumor cells.

Myopericytoma is often misdiagnosed as sarcoma [15]; therefore, we consider it very important to perform appropriate histological and immunohistochemical tests and, if necessary, seek a second opinion from an institution in which rare cases of soft tissue sarcoma are seen more frequently [3]. Adequate sampling is essential for accurate diagnosis. The grading of the tumor is also a significant factor influencing treatment (grade 1 tumors require surgical excision; grade 2 requires additional chemo/radiotherapy based on an individual assessment; and for grade 3, combined treatment is recommended) [3].

To date, there has been no clear recommendation published on the treatment of malignant myopericytomas. In most case reports, complete surgical resection was considered as the primary choice of treatment. The addition of chemotherapy and/or radiation therapy was considered on an individual scale. Bianca N et al. reported the case of a 56-year-old man, who, despite chemotherapy, did not experience a significant tumor response, so surgical resection was performed (R0, N0), after which the disease did not return during the 5‑year follow-up period [9].

In our case, surgical resection was not an option due to the inner ear localization of the tumor. Intracranial myopericytoma is rarely mentioned in the literature: in the article published by Rousseau A et al., three cases were reported, all involving females in their 50s diagnosed with benign myopericytoma [16]. Literature data do not support the effectiveness of chemotherapy for malignant myopericytoma. However, given the young age of our patient, the unfeasible surgical resection, and the malignant nature of the disease, systemic chemotherapy was the final choice of therapy.

There is also limited international data available on radiation therapy. In the literature reviewed, we found only a small number of articles mentioning radiation treatment with a specified dose (Table 1). McMenamin et al. described 5 adult patients, one of whom received postoperative radiation therapy (detailed data on radiation therapy were not provided) [5]. Lavezzo et al. reported a case of benign intracranial myopericytoma in which Gamma Knife (Elekta, Stockholm, Sweden) radiotherapy was performed due to the progressive tumor detected in postoperative images. After 2 years, the lesion had completely regressed on follow-up scans [17]. Cox et al. presented the case of a 70-year-old man with a malignant spinal myopericytoma. Following surgical resection, radiation therapy was applied [6]. Agrawal N et al. published the case of a 50-year-old woman with spinal myopericytoma receiving 5 weeks of radiotherapy. No recurrence was observed at the end of the 32-month follow-up period [10]. Binesh et al. reported the case of a 15-year-old girl with a malignant myopericytoma in her left shoulder. Surgical excision was performed, and because microscopic residual disease could not be excluded by histological examination, she received six cycles of postoperative chemotherapy followed by radiation therapy with a linear accelerator; 18 months after the treatment, her follow-up MRI was negative [13].

**Table 1:** Summary of cases treated with radiotherapy

References	Sex (age)	Location	Tumor size	Treatment	Radiation technique	Dosage	FX	GTV	Outcome
McMenamin et al. [5]	Male (46)	Left thigh	130 mm	Surgery and radiotherapy	No detailed info	No detailed info	No detailed info	No detailed info	Multiple metastasis at 6 months, DOD at 7 months
Cox et al. [6]	Male (70)	Thoracic spine	1.9 cm × 0.9 cm × 0.4 cm	Surgery and radiotherapy	4- to 5‑week course of radiotherapy	Total dose of 45 Gy	No detailed info	No detailed info	CR at 32 months
Agrawal et al. [10]	Female (50)	Thoracic spine	No detailed info	Surgery and radiotherapy	5 weeks of radiotherapy	Total dose of 45 Gy	No detailed info	No detailed info	CR at 32 months
Binesh et al. [13]	Female (15)	Left shoulder	4.4 cm × 5.2 cm	Surgery, chemotherapy, radiotherapy	With linear accelerator	Total dose of 54 Gy with 18-MV photons	In 30 fractions	No detailed info	CR at 18 months
Lavezzo et al. [17]	Female (19)	Posterior cranial fossa	2.75 cm^3^	Surgery and radiotherapy	Gamma Knife radiotherapy	Prescription dose: 15 Gy, prescription isodose: 50%, max. dose: 30 Gy, average dose 21.4 Gy, integral dose: 58.7 mJ	In 15 fractions	3.88 cm^3^	CR at 24 months

*DOD* date of death, *CR* complete remission, *Gy* gray, *mJ* millijoule, *FX* fraction, *GTV* gross tumour volume

In our case, we decided to perform CyberKnife treatment due to the young age of the patient and the location of the tumor. CyberKnife technology has been available in Hungary since the end of 2017, at the National Institute of Oncology, Budapest. The advantage of the CyberKnife technology is that the spatial accuracy of dose delivery is below 1 mm, allowing the radiation to be precisely focused on the tumor while minimizing the radiation exposure of the surrounding healthy tissues. Compared to conventional radiation therapy, its biggest benefit is that a much higher tumor-killing dose can be delivered to a relatively small volume, while keeping the dose to surrounding tissues low. In our case, it seemed optimal to choose this type of radiation therapy method, because the tumor originated in the region of the temporal bone, which is surrounded by sensitive organs.

Our patient received a total of 35 Gy, administered in 5 fractions of 7 Gy, which is approximately equivalent to a total dose of 50 Gy with conventional 25 × 2 Gy fractionation. We also considered this type of radiation treatment to be favorable because it can usually be performed on an outpatient basis. While conventional radiotherapy can take up to 5–8 weeks, a CyberKnife treatment takes 1–5 sessions. Additionally, the duration of one treatment (fraction) varies from person to person, but usually one session lasts between 20 and 45 min. This was an important consideration for our patient because, given her young age, she would not have been able to cooperate properly during the radiation sessions. She was the first child in Hungary to undergo CyberKnife radiotherapy performed under general anesthesia, which posed an additional technical challenge, as the CyberKnife apparatus moves around the patient’s head during treatment while the child is receiving intravenous anesthesia. From this perspective, it was also advantageous that the child received the full dose of radiation in as few sessions as possible.

The control MRI images of our patient indicated that the effects of the targeted irradiation were not immediately visible. However, it is generally observed that tumor growth halts following treatment. Over time, there is a gradual reduction in tumor size as the tumor tissue undergoes necrosis.

In terms of side effects, the treatment has proven to be well tolerated. Our patient experienced no short-term adverse effects, and only minimal long-term post-irradiation changes were noted in the follow-up MRI. These changes included a reduction in the volume of the nasal and pharyngeal tonsils and the submandibular salivary gland. Overall, the treatment appears to be effective, with a favorable safety profile.

## Conclusion

The significance of this case lies in the fact that our patient was the first child in Hungary to undergo CyberKnife stereotactic radiotherapy under general anesthesia. Adequate local tumor control was achieved by a dose of 5 × 7 Gy despite the aggressive clinical behavior. Stereotactic treatment allowed for maximal protection of adjacent organs and structures with minimal side effects. Overall, in our case, the combination of systemic chemotherapy and CyberKnife radiotherapy proved to be effective in the treatment of malignant myopericytoma. Given the limited data available in the literature, it is essential to carefully weigh up the potential risks and benefits of each therapeutic option—particularly in the context of pediatric radiation oncology. A multidisciplinary approach, continuous review of emerging treatment modalities, and individualized planning are strongly recommended to ensure optimal patient care. The careful selection of the radiation method plays an exceptional role in young children, especially in very sensitive tumor locations like the inner ear. Our case report is unique in terms of the patient age, tumor location, and the choice of local treatment. However, due to the rarity of the malignant myopericytoma at such a young age, no general conclusions can be drawn from our results.

## Abbreviations

CEVAIE: Carboplatin, epirubicin, vincristine, ifosfamide, actinomycin‑D, etoposide
CR: Complete remission
CT: Computed tomography
CTV: Clinical target volume
CWS: Cooperative Weichteilsarkom Study
EpSSG: European Paediatric Soft Tissue Sarcoma Study Group
FISH: Fluorescence in situ hybridization
GKRS: Gamma Knife Radiosurgery
GTV: Gross tumor volume
MLC: Multileaf collimator
MRI: Magnetic resonance imaging
OTE: Oral trofosfamide + etoposide
OTI: Oral trofosfamide + idarubicin
RT: Radiation therapy
SRS: Stereotactic radiosurgery
VAC: Vincristine, actinomycin‑D, cyclophosphamide
WHO: World Health Organization
